# Two-Channel Detecting Sensor with Signal Cross-Correlation for FTIR Instruments

**DOI:** 10.3390/s22228919

**Published:** 2022-11-18

**Authors:** Krzysztof Achtenberg, Janusz Mikołajczyk, Zbigniew Bielecki

**Affiliations:** Institute of Optoelectronics, Military University of Technology, 00-908 Warsaw, Poland

**Keywords:** spectroscopy, FTIR, cross-correlation, SNR, photoreceiver

## Abstract

This paper’s purpose was to demonstrate a performance of a novel approach in a low-noise optical sensor for an FTIR spectrometer. Methods: Compared to the standard FTIR detection setup, our sensor ensures a higher signal-to-noise ratio (SNR) and lower signal standard deviation by reducing the uncorrelated noise components (e.g., thermal and 1/*f* noises of the detection module). Its construction is based on two-channel detection modules and a processing unit with implemented cross-correlation signal analyses. Each module was built of LWIR HgCdTe photodiodes and low-noise transimpedance amplifiers. Results: the experiments demonstrated a decrease in a signal standard deviation of about 1.7 times with a 10 dB-improvement in the SNR. Conclusion: this result indicates our sensor’s main benefit, especially in registered “weak” and noisy interferograms.

## 1. Introduction

Fourier Transform Infrared spectrometers (FTIR) are widely used for chemical or optical compound analyses. It is a universal research tool to identify pure substances, mixtures, impurities, or composites [1]. More practical FTIR applications include the analysis of pharmaceuticals, food, semiconductors, polymers, plastics, biological specimens, the environment, and hazardous materials [2,3,4,5,6]. The measurement virtues depend mainly on the performances of the applied FTIR instrument, e.g., resolution, precision, and floor (background) noise.

In practice, many noise sources essentially affect both spectral and power domains of the registered spectra. Usually, the quality of the spectral analysis is determined by the ratio of signal-to-noise (*SNR*) [7,8,9]. Its value depends on operation scenarios and FTIR instrument configuration and can be expressed by [10]:

(1) $$SNR=\frac{U_{V}\cdot \Theta \cdot \Delta \upsilon \cdot t^{\frac{1}{2}}\cdot \xi }{NEP}$$

where Θ—throughput, *ξ*—efficiency, Δ*ν*—resolution, *t*—measurement time, *NEP*—noise equivalent power of detection module, and *U_V_*—detector output signal. Equation (1) describes the main spectroscopic trading rule specifying *SNR*, resolution, and measurement–time relationships. 

If detection module noise is predominant, spectral *SNR* increases proportionally with the FTIR throughput. This parameter depends on the area of the projected mirror and spectral resolving power (ratio of the maximum analysed wavenumber and spectral resolution). It can also be related to a collimating system’s divergence angle, aperture, and focal length. 

In an ideal setup, the total noise is substantially limited by the noise of the used detection module regardless of the signal level. The simplest and most common method to increase the *SNR* is to perform a series of measurements and average the results. For the coherent signals and the random noise, the *SNR* increases with the square root of the number of averaged interferograms. However, if the noise level of the detection module is below the resolution of the used analogue-to-digital converter (ADC) (below the least significant bit of the ADC), this procedure does not improve the *SNR*, and quantisation noise in the spectrum is noticed. Applying a gain-ranging amplifier with ADC minimises this noise’s impact by setting the interferogram’s dynamic range. In this configuration, the gain is switched in the time interval between sample points. Hirschfeld [11] applied gain ranging as a series of steps approximating the interferogram envelope. For the registered interferogram, the *SNR^I^* is given by [12]:

(2) $$SNR_{FTIR}^{I}\propto {\left(\frac{T}{N_{I}}\right)}^{1/2},$$

where *T* is the measurement time and *N_I_* is the number of interferogram points determined by *N_I_ = x_max_/d_s_* (*x_max_*—the maximum mirror retardation distance, *d_s_*—the sampling period). 

The output signal of the detection module depends on parameters individually defined for a specific model of FTIR spectrometer, e.g., transmission and geometry of optics, detector’s characteristics, the speed of the moving mirror, and signal bandwidth. The average radiation power registered by FTIR’s detector equals:

(3) $$P_{I}=\frac{V}{GR},$$

where *V* is the interferogram wing’s mean signal, *G* is the gain of the detection module’s amplifier, and *R* is the photodetector responsivity. In the best practice, the *SNR* value of FTIR is optimised to provide a level defined by the minimum noise level of the detection module:

(4) $$SNR_{detection}^{I}=\frac{P_{I}}{N_{deteciion}},$$

where *N_detection_* is the power spectral density (PSD) of the detection module noise (both photodetector and amplifier). 

During conversion to a spectral area, the spectral signal-to-noise ratio (*SNR^S^*) increases by:

(5) $$SNR^{S}=SNR^{I}\times N_{I}^{1/2}.$$

The value of *SNR^S^* can also be defined by:

(6) $$SNR^{S}=SNR^{I}\times N_{I}^{1/2}\times \frac{\rho \left(\upsilon \right)}{\rho_{total}},$$

where *ρ*(υ) and *ρ_total_* are the spectral power density at the analysed wavenumbers and the total spectral power, respectively. 

The FTIR spectrometer operation is accepted if the spectral *SNR* is not below 10^5^. We can determine the spectral noise *N*(*ν*) during the ratio measurement of two spectra (*T_a_* and *T_b_*) by calculating:

(7) $$N\left(\upsilon \right)=1-\frac{T_{a}\left(\upsilon \right)}{T_{b}\left(\upsilon \right)}.$$

**Figure 1:**
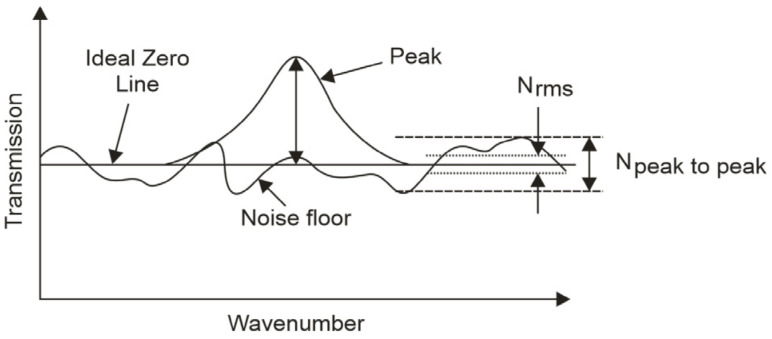
Noise influence analyses in transmission characteristics.

Its level corresponds to the peak-to-peak value (*N_p-p_*—difference between the maximum and minimum values) or the root–mean–square value (*N_rms_*—a standard deviation of all data points) (Figure 1). *N*(ν) equals 0 in the tested wavelength range for a noiseless device. Usually, the FTIR manufacturer’s datasheet specifies the noise feature by a *100% line* defined by 100 × *T*_a_/*T*_b_. Considering values of the spectral noise *N_rms_*, the spectral *SNR^S^* can also be described by:

(8) $$SNR^{s}=\frac{1}{N_{rms}}.$$

A more practical method of *SNR* determination analyses the shape of the registered transmission spectrum with the peak of spectral transmission and the floor noise. In this case, the noise level can be defined at the base of this spectrum. In this situation, the *SNR* equals:

(9) $$SNR=\frac{µ\left(\upsilon \right)}{rms\left[T\left(\upsilon \right)-µ\left(\upsilon \right)\right],}$$

where µ is the ideal mean (“noiseless”) transmission spectrum and *T* is the measured one. This equation can be transformed into:

(10) $$SNR=\frac{µ\left(\upsilon \right)}{µ\left(\upsilon \right)rms\left(\frac{T\left(\upsilon \right)}{\mu \left(\upsilon \right)}-1\right)}=\frac{1}{rms\left(\frac{T\left(\upsilon \right)}{\mu \left(\upsilon \right)}-1\right)}.$$

We can visualise the detection noise impact on spectral measurements with an FTIR spectrometer based on the transmission characteristics presented in Figure 1. The detection limit and the noise floor define the detection resolution and the spread of noise amplitude, respectively. The same noise parameters are a critical issue in the measurements of transmission characteristics and absorption lines.

Usually, the primary noise sources of detection are a photodetector, its power supply, and a preamplifier [13]. Currently, much work is being carried out to obtain ultra-low-noise photoreceivers. We ensured the maximum *SNR* detection value based on photodetectors’ noise models and read-out electronics. Finally, it allows the selection of the detector’s operating point and electronics configuration, determining the actual performance of the detection module, e.g., operating temperature, biasing voltage, signal gain, and bandwidth. It is intended that the noise of the detection module is determined solely by the detector one [14]. However, detector technology development poses a significant challenge for designing “noiseless” read-out electronics. In papers [15,16,17,18], we analysed some advanced development methods to minimise the noise influence of such electronics. 

However, the presented paper describes a novel approach in this field of knowledge because it defines new possibilities for FTIR spectroscopy with a unique low-noise optical sensor. The literature analysis showed that many methods of increasing the SNR ratio have already been developed [12,19,20,21,22]. For the detection module, a common way was to select a low-noise detector and reduce its operating temperature [23]. A more complex method applies a dual-beam FTIR spectrometer with a single detection module [23,24,25]. It minimises the common background and decreases the dynamic range of the measured signal. The obtained reduction of the dual-beam interferogram amplitude decreases quantisation noise contribution by effectively using the ADC dynamic range. For example, the *SNR* of the dual-beam systems described in [23,25] were a factor of 9 and 6 better than that of the single-beam one. 

However, none of the referenced papers has considered the possibility of using the described configuration of the detection module and procedure of signal analyses. To the best of the authors’ knowledge, this work demonstrates, for the first time, that:

- A two-channel detection module with cross-correlation can be used as an optical detection unit in an FTIR setup;
- The sensor with signal cross-correlation improves the signal-to-noise ratio of the FTIR spectrometer and is more effective than spectra averaging.

## 2. Materials and Methods

### 2.1. Experimental Design

**Figure 2:**
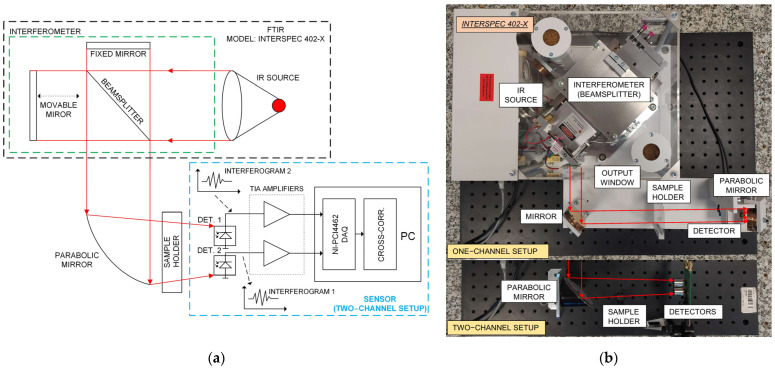
Scheme (**a**) and photo (**b**) of an FTIR spectrometer with a designed sensor.

The proposed method is based on a two-channel optical detection with a signal cross-correlation approach. Although this is a known method, it has not yet been applied to decrease floor noise in an FTIR spectrometer. The core of this method is that the measurements are performed simultaneously with two similar detection channels [26]. Based on the statistical character of signals, spectrum extraction of the valid signal from one noise is possible [27]. Figure 2 presents a scheme and a photo of an FTIR spectrometer with an optical sensor consisting of two photodetectors, read-out electronics, and a signal processing unit with implemented cross-correlation algorithm (to calculate a cross-power spectrum density—CPSD). 

The cross-correlation technique is based on the simultaneous measurement of two signals, *V*_1_*(t)* and *V*_2_*(t)* [28,29]. These signals contain the common signal *V_s_(t)* and uncorrelated noise components of *V_n_*_1_*(t)* and *V_n_*_2_*(t)*:

(11) $$V_{1}\left(t\right)=V_{s}\left(t\right)+V_{n1}\left(t\right),$$

(12) $$V_{2}\left(t\right)=V_{s}\left(t\right)+V_{n2}\left(t\right).$$

The cross-correlated spectrum *S*_12_*(f)* is calculated by multiplying the Fourier transforms of the first signal *F*[*V*_1_(*t*)] by a complex conjugate of the second one *F*[*V*_2_(*t*)]:

(13) $$S_{12}\left(f\right)=F\left[V_{s}\left(t\right)\right]\times F{\left[V_{2}\left(t\right)\right]}^{*}.$$

The procedure is averaged, and the uncorrelated components of these signals are reduced:

(14) $$S_{12}{\left(f\right)}_{avg}=\frac{\sum_{n=1}^{N}S\left(f\right)}{N}.\;$$

However, there is a need to perform more averages to obtain smooth spectra compared to FTIR standard averaging operated with a single detector. 

The result of these calculations has the form of the frequency spectrum. This spectrum is transformed into the wavenumber domain in FTIR spectrometers by applying a reference laser with a unique wavelength. The laser beam travels the same optical path through the interferometer generating an interferogram as a sinusoidal signal. This signal determines the interferometer displacement and defines the wavenumber [30]. If there is no possibility to apply reference laser signals, the common practice is to use some special calibration samples with a well-defined spectral position of absorption lines [31]. 

The described sensor is mounted into the radiation path of the FTIR spectrometer, and the FTIR output beam is focused on its photodetectors using an off-axis parabolic mirror. A high-resolution data acquisition card and PC software digitise the photodetectors output signals and perform calculations. 

The designed FTIR setup requires the application of two photodetectors with similar spectral characteristics of relative responsivity. Any differences in these characteristics cause significant distortions in the results of the cross-correlation procedure. These differences can generate a high-level spread of cross-spectrum products in some wavelength ranges, leading to adulterations in the final results. 

In a classical FTIR detection setup, the parabolic mirror focuses optical radiation on an active area of a single detector. In the case of the described sensor, irradiation of an active surface of two separate detectors causes a decrease in the registered optical signal. Theoretically, better technology can use a two-segment detector in which registered optical power depends on the chip dimensions. Each segment detects a part power of FTIR light. 

### 2.2. Sensor Design

There are some critical issues in analysing a sensor construction. For example, the applied detectors should have high spectral detectivity and broad signal bandwidth. These parameters define, e.g., the *SNR* level and the limitation of radiation analyses in the high wavenumber region, respectively. The noise level also depends on a detector’s read-out electronics, and some aspects of its low-noise design were described in the literature [32,33].

### 2.3. Photodetectors

**Figure 3:**
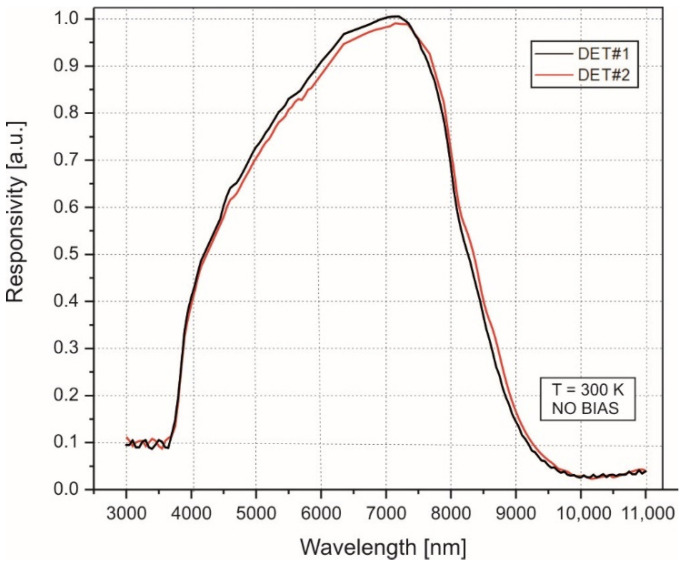
The relative responsivity of the applied HgCdTe photodiodes at 300 K.

The applied HgCdTe heterostructure photodiodes were manufactured during the same technology process. These photodiodes with an optical area of 0.5 × 0.5 mm^2^ (TO-8 package) operated at room temperature with no biasing voltage [34,35]. Their similar relative responsivity has a peak value at a wavelength of *λ_peak_* = 7 µm (Figure 3).

### 2.4. Read-Out Electronics

**Figure 4:**
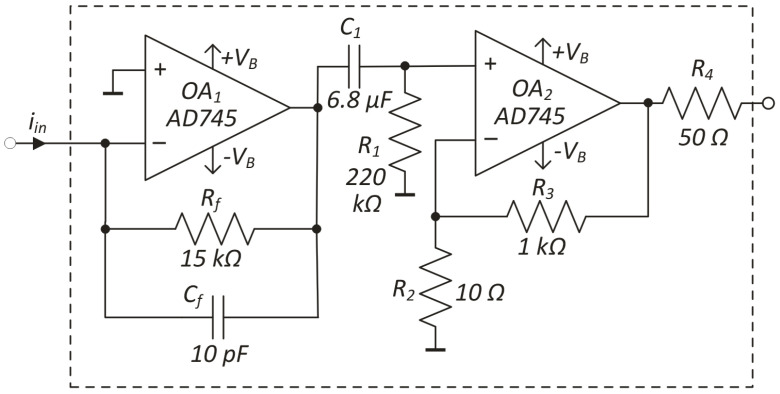
Scheme of a single channel of the designed read-out electronics.

The constructed read-out electronics consist of two identical signal channels based on the typical opamps configurations. The photocurrent signal from the detector is converted by a transimpedance amplifier (TIA) and gained by a voltage amplifier (VA). Figure 4 presents a schematic of the single signal channel. Its key parameters are a transimpedance of 15 × 10^5^ V/A and −3 dB bandwidth in the 0.1 Hz–5 kHz range. 

The channel output signals were connected to a PC-mounted data acquisition card from the National Instruments, model PCI-4462. It has four simultaneous sampling inputs with built-in anti-aliasing filters providing a 24-bit resolution and a 204.8 kS/s sampling frequency. The digitised interferograms are stored in the PC and transformed into the spectrum domain (power spectrum density and cross-power spectrum density). Finally, the spectrum can be expressed in the wavenumbers or wavelengths domain using, e.g., calibrated absorbers.

## 3. Experiment Results

### 3.1. FTIR Spectrometer with a Classical Detection Setup

**Figure 5:**
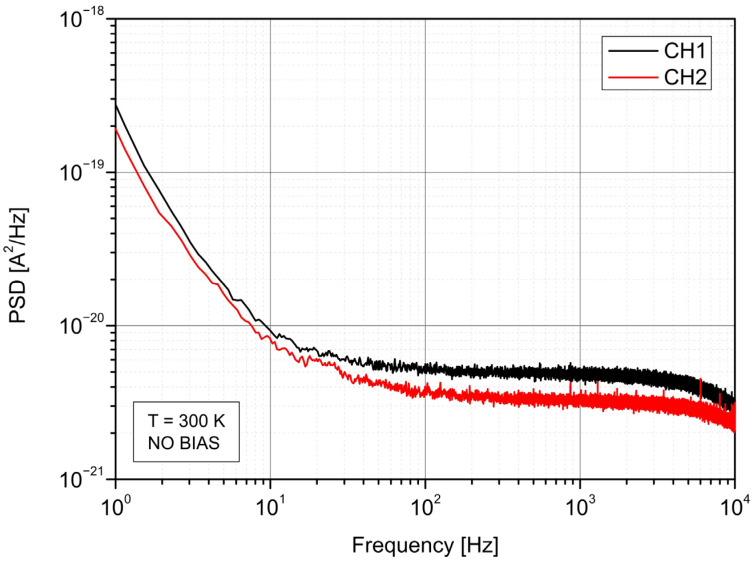
Spectral density of noise power of the designed channels.

In the first step of the work, the noise power spectral density of two signal channels (CH1 and CH2) was measured separately. It allows determining the fundamental limit considering floor noise for each of them. Figure 5 presents the measured noise characteristics in which 1/*f* and thermal noise regions can be defined. In comparison, the noise limit of the CH1 detection channel is a little worse than CH2’s, resulting from different values of the detectors’ resistances. The 3.2 × 10^−21^ A^2^/Hz and 5 × 10^−21^ A^2^/Hz PSD values of thermal noise at 1 kHz are consistent with the photodiodes resistances. 

**Figure 6:**
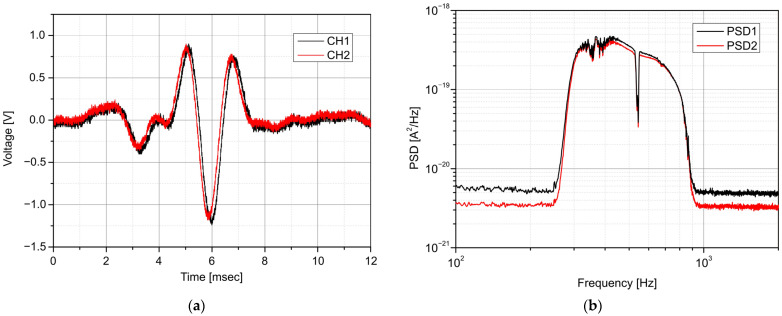
The interferograms registered separately by CH1 channel and CH2 channel (**a**) and their PSD (**b**) without any absorbing sample placed in FTIR optical path.

Both channels were placed into an optical beam of an FTIR spectrometer, model Interspec 402-X [36]. The position of the photodiodes was adjusted to have the same amplitude of the registered signals. Figure 6 presents an example of interferograms and their power spectral density. The shape of the recorded spectra is determined by the spectral characteristics of the FTIR radiation source, attenuation of an optical path (optics and air transmission), and the spectral responsivity of HgCdTe photodiodes (without any absorbing sample).

The registered interferograms were characterised by a low amplitude difference and a phase shift (time delay) generated by photodetectors and their read-out electronics (different signal phase-frequency response). A difference in the noise levels of the used detectors was also observed. In practice, it is impossible to manufacture two detecting structures with perfectly even parameters, e.g., responsivity and noise. 

### 3.2. FTIR Spectrometer with the Designed Sensor

The preliminary experiment defined the performance of cross-power spectrum analyses in an FTIR spectrometer. The influence of some key factors (the FFT algorithm parameters and averaging time) on signal conditioning, acquisition, or transformation was also defined. These analyses are based on signals registered using a classical FTIR setup (with one detector) and the designed two-channel system with the CPSD procedure. The classical setup uses autocorrelation of the one detector output signal to determine its PSD (*S*_11_). In contrast, the CPSD is simultaneously based on signals registered by both detectors (*S*_12_). 

**Figure 7:**
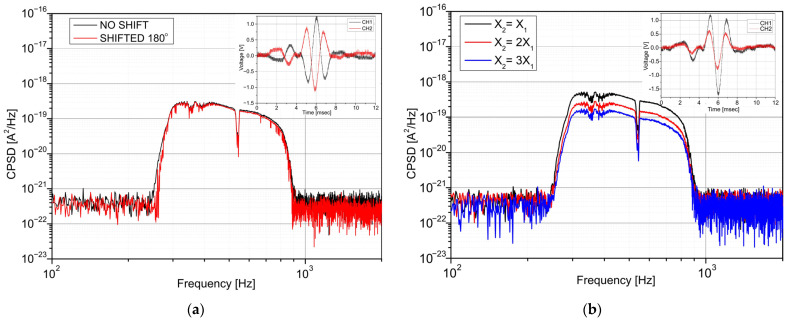
Cross-spectra of two interferograms with different phase shifts (**a**) and amplitudes (**b**).

To validate the performance of the measurement procedure, the influence of the phase shift and amplitude differences of the two detectors’ signals on the CPSD procedure was analysed. Figure 7a,b illustrate interferograms and their cross-spectra for two values of phase shifts (0° and 180°) using an analogue phase shifter and different amplitude ratios (1/1, 1/2, 1/3) setting the optical beam alignment. The phase shift was provided at the amplifier output using an opamp-based analogue circuit described in [37].

The interferograms’ phase shift (time delay) does not influence the cross-spectra. A significant change in cross-spectra was observed based on the results for different amplitude ratios. The highest level of CPSD was for the interferograms with the same amplitudes. 

**Figure 8:**
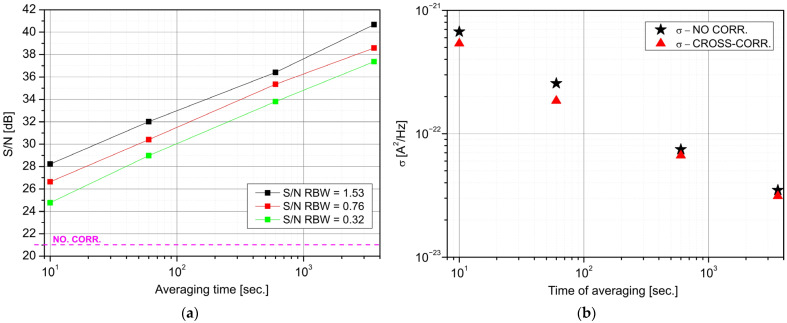
Influence of averaging time on *SNR* values for different RBWs (**a**) and noise spread (standard deviation) (**b**).

The cross spectra were also determined for different averaging times (10 s, 60 s, 600 s, and 3600 s). These spectra were compared with averaged PSD for one detector’s setup (NO CORR.—green line) (Figure 8a). We observed a noticeable decrease in the floor noise level with increasing averaging time. However, there is a slight reduction in the spread of noise amplitude. Noise influence can be spectrally filtrated by the FFT procedure, defined by its resolution bandwidth (RBW). The resolution is determined by the number of data points and sampling frequency. Figure 8b presents CPSD values for different RBW and averaging times. The better RBW corresponds to a high resolution in the spectrum, while for limited stored data, it also limits the maximal number of averaging. There are also observed differences in the theoretical analyses of RBW’s influence on noise reduction with the experimental results. The measured noise level is higher because of “real” signal parameters (e.g., correlated components of signal) and “non-ideal” applied devices (e.g., transimpedance gain characteristics). 

**Figure 9:**
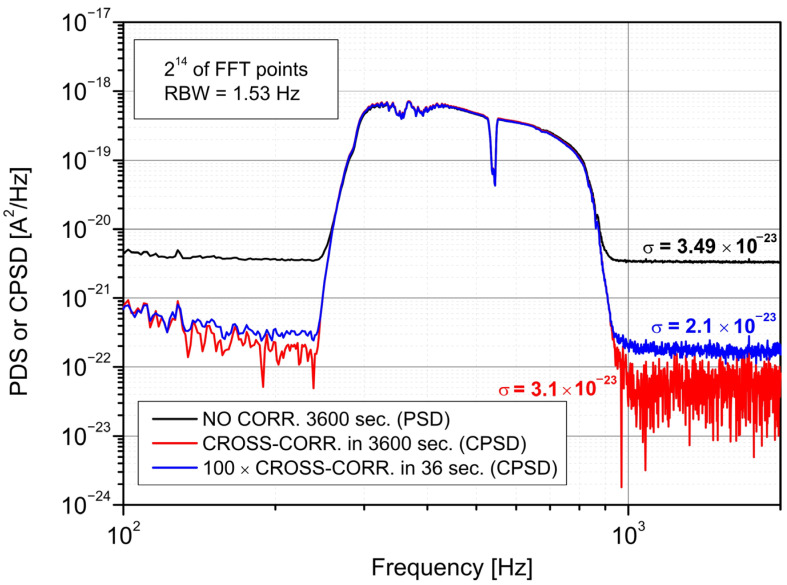
The spectra after cross-spectra averaging.

Figure 9 presents the influence of a linear averaging of segmented data points (1-h data) on registered spectra. However, this procedure “smooths” the noise spectrum and increases the floor noise. It is due to the shorter calculated time of each cross-spectrum. 

It was shown that the application of data segmentation (averaging many cross-spectra) compromises the reduction of floor noise or standard deviation σ. However, the proposed averaging provides a better standard deviation and floor noise than the single detector results. Using this method, the σ was about 1.7 times lower, with a 10 dB improvement of the *SNR*.

### 3.3. Practical Validation of the Designed FTIR Setup

**Figure 10:**
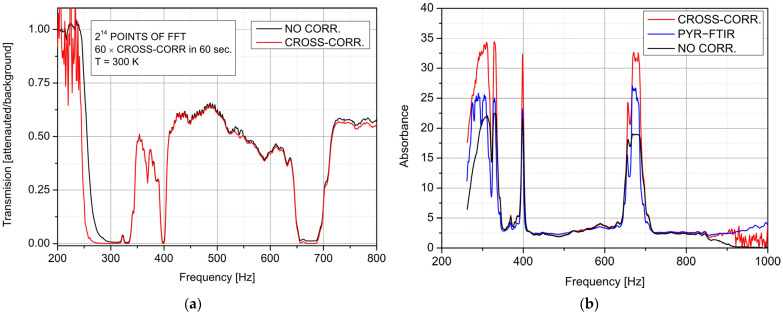
Comparison of the foil spectral transmission registered with one- (NO CORR.) and two-(CROSS-CORR.) detection channels. (**a**) The foil absorbance determined with the standard pyroelectric detection module (PYR–FTIR) and designed sensor in a two-channel (CROSS-CORR.) and a single-channel (NO CORR.) configuration (**b**).

The practical advantages of the designed sensor in FTIR spectra measurement were defined during the transmission investigation of a foil sample (Polyvinyl Chloride containing a plasticiser). The transmission was determined using the same FTIR spectrometer (model Interspec 402-X, Interspectrum OU, Tartumaa, Estonia) equipped with the sensor but using auto-correlation of the signal registered by one channel and cross-correlation with both channels. The RBW was set to 1.53 Hz by *f_s_* = 25 kS/s and 2^14^ FFT points during that research. Figure 10a presents a determined transmission of the sample with the designed sensor. These characteristics were drawn in the frequency domain, comparing the results obtained in the two-detector (CROSS-CORR.) and one-detector (NO CORR.) configuration of FTIR detection to simplify analyses. The HgCdTe detection modules were adjusted into the optical path to ensure the amplitude difference of interferograms below 1%.

The sensor collected data for 1 h. The shape of the foil transmission characteristics is similar, but the most remarkable differences are in the low-frequency range. We observe a large spread of transmission values resulting from the division of two low signals corresponding to the noise level after the cross-correlation operation and an expansion analysis range with the 1/*f* noise decrease of the read-out electronics. Low transmission in the 650 Hz–700 Hz range shows a better detection limit of the “cross-correlation sensor”. It is an essential virtue in spectral absorption line measurement in which both ultra-low level and ultra-low spread of floor noise are required. These parameters directly influence the detection limit and the resolution of spectral analyses. That is why further analyses concern the foil absorbance. The results were compared with the data determined by the FTIR spectrometer equipped with its standard detection module with a pyroelectric photodetector (PYR–FTIR). Figure 10b compares the spectral absorbances of the foil registered by the PYR detection module and the designed sensor (with two detection channels—CROSS-CORR., and a single one—NO CORR.). The application of the designed sensor in FTIR spectrometry provides better dynamic sensitivity compared to the standard setup with the PYR–FTIR module. We can distinguish absorption lines more clearly using our sensor. This virtue is essential, e.g., during analyses of the spectral blocking filters or optical high-attenuated compounds.

## 4. Conclusions

The new approach to analysing and processing signals in FTIR instruments was described in this paper. The developed two-channel sensor can be used mutually with others dedicated to improving *SNR*, e.g., detector cooling. Its operation is based on signal cross-correlation that minimises thermal noise and reduces the 1/*f* one, which is typically problematic in the standard configuration when uncooled or biased detectors are used. The proposed sensor can recover the interferogram signals from unexpected noise. Most noise sources in the described sensor configuration are uncorrelated, so cross-correlation is a perfect algorithm to reduce them. The paper also analysed the advantages and disadvantages of cross-correlation operation considering photodetector characteristics and a two-channel detection setup.

A direct comparison of results from the same averaging time speaks for the cross-correlation procedure. The obtained spectra were characterised by a standard deviation about 1.7 times lower, with a 10 dB improvement of the *SNR* value comparing a “single-channel” classical setup. For FTIR systems, the designed sensor ensures a wider wavelength span and improves measurement precision in the case of noisy or low-amplitude interferograms. It becomes a practical tool for detecting and selecting the absorption lines of the analysed compounds.
